# MAST Kinases’ Function and Regulation: Insights from Structural Modeling and Disease Mutations

**DOI:** 10.3390/biomedicines13040925

**Published:** 2025-04-09

**Authors:** Michael C. Lemke, Nithin R. Avala, Michael T. Rader, Stefan R. Hargett, Daniel S. Lank, Brandon D. Seltzer, Thurl E. Harris

**Affiliations:** Department of Pharmacology, University of Virginia, Charlottesville, VA 22903, USA; mcl4ph@virginia.edu (M.C.L.);

**Keywords:** MAST kinase, 14-3-3, mutations, AlphaFold, DUF1908

## Abstract

**Background/Objectives**: The MAST kinases are ancient AGC kinases associated with many human diseases, such as cancer, diabetes, and neurodevelopmental disorders. We set out to describe the origins and diversification of MAST kinases from a structural and bioinformatic perspective to inform future research directions. **Methods**: We investigated MAST-lineage kinases using database and sequence analysis. We also estimate the functional consequences of disease point mutations on protein stability by integrating predictive algorithms and AlphaFold. **Results**: Higher-order organisms often have multiple MASTs and a single MASTL kinase. MAST proteins conserve an AGC kinase domain, a domain of unknown function 1908 (DUF), and a PDZ binding domain. *D. discoideum* contains MAST kinase-like proteins that exhibit a characteristic insertion within the T-loop but do not conserve DUF or PDZ domains. While the DUF domain is conserved in plants, the PDZ domain is not. The four mammalian MASTs demonstrate tissue expression heterogeneity by mRNA and protein. MAST1-4 are likely regulated by 14-3-3 proteins based on interactome data and in silico predictions. Comparative ΔΔG estimation identified that MAST1-L232P and G522E mutations are likely destabilizing. **Conclusions**: We conclude that MAST and MASTL kinases diverged from the primordial MAST, which likely operated in both biological niches. The number of MAST paralogs then expanded to the heterogeneous subfamily seen in mammals that are all likely regulated by 14-3-3 protein interaction. The reported pathogenic mutations in MASTs primarily represent alterations to post-translational modification topology in the DUF and kinase domains. Our report outlines a computational basis for future work in MAST kinase regulation and drug discovery.

## 1. Introduction

The necessity and utility of protein kinases have driven the evolution from primordial origins to the highly diverse superfamily of proteins that we now appreciate in the eukaryotic genome [1]. Researchers and clinicians alike have long investigated these enzymes, as they are powerful biological signal integration units with pervasive downstream effects that are often dysregulated in metabolic disease and oncogenic transformation [2]. Significant progress has been made in understanding kinase pharmacology since the first kinase inhibitor was approved over twenty years ago [3]. However, even though the majority of kinases preferentially phosphorylate serine/threonine residues [4], serine/threonine kinase inhibition has struggled to make it through clinical trials compared to receptor tyrosine kinase inhibition, primarily due to issues with selectivity and disease resistance [5]. Although identifying effective inhibitors of serine/threonine kinases is an active field of research that constantly develops new targeting strategies [6,7], current investigations of the biochemistry of these kinases are still limited by pervasive annotation bias. This phenomenon, referred to as the ‘streetlight effect’, leaves many proteins understudied and uncharacterized [8,9].

The ‘AGC’ kinases are a family of serine/threonine kinases with catalytic cores homologous to cAMP-dependent protein kinase (PKA), cGMP-dependent protein kinase (PKG), and protein kinase C (PKC) (AGCs) [10]. Although many of these kinases have been extensively studied, the essential functions of some AGC kinases remain obscure [11]. The AGC family includes over sixty individuals subdivided into over twenty subfamilies based on non-catalytic homology [11]. Most AGC kinases possess other subfamily-specific functional domains outside the catalytic core that regulate the functionality of the kinases. The most well-studied AGC kinases, including PKA/G/C, PDK1, S6K, and Akt (PKB), were the first to be identified and are directly involved with both cancer and diabetic disease states [11,12]. However, mutations in many lesser-investigated AGC kinases, such as serum and glucocorticoid-regulated kinase (SGK), nuclear Dbf2-related (NDR) kinases, and microtubule-associated serine/threonine (MAST) kinases, are also closely associated with diseases and inherited conditions [11]. Without a deeper understanding of the biomolecular mechanisms involving the mutated proteins that drive pathogenesis, clinicians are currently limited to treating disease symptoms rather than addressing the causative agent. Therefore, reinforced by coinciding advancements in mass spectrometry, there is a renewed focus on often-ignored members of the proteome at the margins of biochemical properties like size and abundance to provide the next clinical breakthrough [8,9,13,14]. New biological insights into the function of these proteins, such as G-protein-coupled receptors, small GTPases, transcription factors, phosphatases, and kinases, represent promising clinical opportunities [8,15].

## 2. Materials and Methods

### 2.1. Phylogenetic Analysis

Analyses were conducted through MEGA11 [16]. Depiction of whole protein and activation loop lineage divergence was estimated using the maximum likelihood method and JTT matrix-based modeling [17]. Bootstrap consensus trees, compiled from 1000 tree construction replicates, are representative of the evolution of the taxa analyzed. The percentage of trees in which associated taxa clustered together in the bootstrap test is shown next to the related branches [18]. The initial trees for the heuristic search were automatically obtained through the Neighbor-Joining and BioNJ algorithms applied to pairwise distance matrices generated by JTT. The topology with the best log-likelihood values was chosen.

### 2.2. Multiple Sequence Alignment

UniProt accession numbers are indicated when referencing gene names in corresponding figures to indicate sequences analyzed [19]. The domain of each UniProt accession depicted in each graphical representation is outlined in the InterPro database [20]. Sections of overlapping sequences were visualized using Clustal-Omega (EMBL-EBI) [21].

### 2.3. In Silico Structural Modeling

Indicated PDB [22] or AlphaFold [23] structures were loaded in PyMOL (PyMOL Molecular Graphics System, Version 3.0.5 Schrödinger, LLC, New York, NY, USA) for visualization. The super command in PyMOL analyzed the similarity between the indicated structures. The sequence-independent and residue-based dynamic programming alignment was refined through cycle-iterative fit improvements to reject outliers. Root-mean-square deviation (RMSD) values, representing structural similarity, were automatically calculated by PyMOL.

Biomolecular interactions between MAST1 (Q9Y2H9) and 14-3-3β (P31946) were simulated via the open-access AlphaFold3 server [24]. The full-length sequence of MAST1 with or without phospho-Ser161 was entered with two copies of the full-length sequence of the 14-3-3β monomer. The prediction accuracy of the 14-3-3β dimer generated by AlphaFold3 was validated via the superimposition of the solved structure of human 14-3-3β (PDB: 4DNK).

### 2.4. Mutational Consequence Estimation

Predictions and consensus interpretations for single-point mutagenic consequences in indicated MAST isoforms were simulated via the free-for-public-use websites Variant Effect oN Structure [25,26] (VENUS, https://venus.cmd.ox.ac.uk/venus, accessed on 29 December 2024) and DDMut (https://biosig.lab.uq.edu.au/ddmut/, accessed on 29 November 2024) [27]. Each online deep-learning-based modeling software was used to estimate changes in Gibbs free energy (ΔΔG, kcal/mol) of point mutations in a protein structure. VENUS ΔΔG (calculated by Mut ΔG–WT ΔG) and DDMut -ΔΔG values (calculated by WT ΔG–Mut ΔG) >2 were considered destabilizing (less thermodynamically favored) and <−2 stabilizing (thermodynamically favored).

## 3. The Origins and Divergence of MAST Kinases

The MAST kinases are one such under-investigated subfamily within the AGC class of serine/threonine kinases that have garnered a fair amount of recent interest [28]. The MAST kinases have been implicated as drivers of many human diseases, such as cancer, metabolic disease, and neuronal disorders [28]. These kinases are highly conserved across nearly all eukaryotic groups, from single-celled protists to complex plants and animals [11,12,29,30]. In the eukaryotic AGC kinome phylogeny, the MAST branch is one of the earliest divergent lineages of AGC kinases [1,31,32,33]. Considering this, the MAST kinases may represent some of the earliest AGC kinase proteoforms.

The number of MAST kinases has increased with multicellularity and organismal complexity in eukaryotic evolutionary history. This is evident from single MAST orthologs existing in species like *C. elegans* (kin-4 [34]) and *D. melanogaster* (drop-out, *dop* [35]), whereas further gene duplication events have led to an expansion of the MAST kinase subfamily beyond the initial division between MAST and MASTL kinases [28]. More recently evolved eukaryotes have four MASTs (MAST1-4) and one MAST-like kinase (MASTL). MASTL is shorter than the others as it only contains the kinase domain, but it is best understood in terms of activation [36,37], regulation [38,39], and substrates [40,41,42,43]. Therefore, this article will focus on the remaining human MASTs, MAST1-4.

MAST1-4 are atypical in the AGC family [11]. These large kinases (~1300–2400 amino acids) possess multiple functional domains outside the AGC kinase core. These include the N-terminal domain of unknown function (DUF1908, InterPro: IPR015022), as well as the C-terminal postsynaptic density protein (PSD95), Drosophila disc-large tumor suppressor (Dlg1), and zonula occludens-1 protein (zo-1) (PDZ) domain. MAST1-4 all have the highly conserved AGC catalytic domain with key structural motifs present in almost all AGC kinases, including the activation segment (T-loop), RD-pocket, hydrophobic motif (HM), and turn motif (TM) [11]. However, the DUF and PDZ domains are unique to the MAST subfamily [11]. Although the structures of the DUF and PDZ domains from many MAST kinases have been solved (PDB: 2M9X, 3PS4, 2KQF/2KYL, 1V9V, 3KHF), it is currently unclear whether either domain contributes to MAST kinase function [11,28]. Rescue studies in MAST-ortholog knockout (*dop*) fruit flies show that all canonical MAST domains are essential [35]. The PDZ domain has been shown to regulate interactions between MAST kinases themselves and other proteins [34,44,45]. The DUF domain is highly modifiable by post-translational modifications (PTMs) [28], and mutations in MAST1-4 in humans are pathogenic for neurological disorders [28,46,47,48].

**Figure 1:**
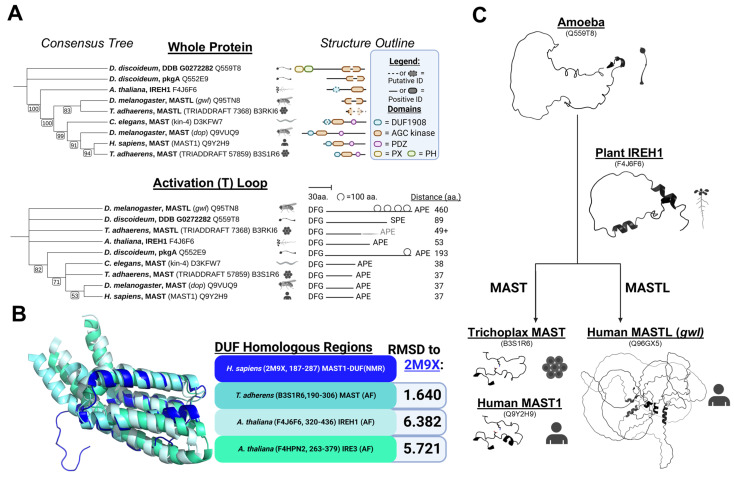
Ancient MAST kinases demonstrate transitional morphology to descendent MAST/MASTL kinases. (**A**) Bootstrap consensus tree generated by MEGA11 for complete annotated sequence and activation (T) loop (defined by DFG to S/APE motifs) of MAST-lineage kinases. The tree image was drawn using the iTOL web server [53]. Taxa identified by gene name and UniProt accession number. The general structural outline of the whole protein and T-loop is visualized to the right of each respective tree. (**B**) Superimposition of indicated regions in MAST lineage species DUF domains. Each species’ protein is color-indicated, and RMSD to MAST1-DUF (2M9X, aa. 187-287 in MAST1 (Q9Y2H9)) is reported. Sources of structures are shown (NMR—Nuclear Magnetic Resonance; AF—AlphaFold). (**C**) Morphology comparison of the transition from MAST/MASTL kinases in protists (Q559T8) and plants (F4J6F6) to Trichoplax (B3S1R6) and humans (Q96GX5; Q9Y2H9). DFG to APE sections within the kinase domain of each model are shown. The graphics were created in BioRender. Lemke, M. (2025) https://BioRender.com/n02b937, accessed on 29 March 2025.

To glean functional insights into the development of MAST kinases, we sought to outline the timeline of MAST kinase evolutionary divergence (Figure 1A). MAST kinase isoforms have been annotated in the genomes of early divergent eukaryotes such as *Trichoplax adhaerens* [49] and *Arabidopsis thaliana* [50]. Slime molds, as represented by *Dictyostelium discoidum*, conserve multiple MAST genes, although some could also be Akt/PKC/NDR homologs [51,52]. The remaining orthologs, DDB G0272282 and pkgA, have MAST-like catalytic domains (specifically, large DFG-APE segments). Although DDB G0272282 does have Phox (PX) and Pleckstrin Homology (PH) domains, which are not conserved in any other MAST lineage kinase, neither DDB G0272282 nor pkgA conserve the DUF or PDZ domain (Figure 1A).

While the earliest identified MAST/MASTL homologs are identifiable only by an AGC kinase domain, the next characteristic MAST domain occurs with the DUF1908 domain. The plant MAST homologs are the incomplete root-hair elongation kinases (IRE, IREH1, IRE3, and IRE4 in *A. thaliana*). IRE kinases do not have a PDZ domain [50]. However, a region previously identified as a putative microtubule-associated domain in IREH1 was predicted to be similar in structure to the C-terminal portions of what is now called the DUF domain in MAST1/2 [50]. This region in IREH1 was sufficient to colocalize IREH1 to the centrosomes when overexpressed in animal cells. We identified similar DUF-homologous regions in the AlphaFold models for IREH1 (F4J6F6) and IRE3 (F4HYG2) (Figure 1B). The DUF domains in MAST kinases are predicted to be partially disordered at the N-terminus but structured in the C-terminal portion in an alpha-helical-barrel conformation (alpha barrel) [28]. When superimposed, we find that the C-terminal alpha barrels of human, *Trichoplax*, and plant IRE DUF domains are strikingly similar (Figure 1B). Therefore, we conclude that plant MASTs conserve the DUF but not the PDZ domain.

MAST isoforms in worms (kin-4), flies (dop), and humans MAST1-4 all share a conserved tri-domain structure consisting of DUF, catalytic, and PDZ domains (Figure 1A) [28]. Early divergent animals, like *Trichoplax adhaerens*, likely exhibit the separation of MAST (B3S1R6) from MASTL (B3RKI6-Fragment) lineages. In contrast, plants conserve MAST homologs with DUF domains but do not have a separate MASTL ortholog [29]. Based on these observations, we hypothesize that the DUF domain emerged just before the divergence of MAST and MASTL lineages and the subsequent appearance of the PDZ domain. Phylogenetic analysis of protist, plant, and animal kinases supports this hypothesis, showing that tri-domain MAST kinases (e.g., B3S1R6, kin-4, *dop*, MAST1) are highly similar and more recently evolved (Figure 1A). In contrast, kinases such as DDB G0272282, pkgA, and IREH1 more closely align with MASTL lineage members (e.g., *gwl*, B3RKI6) but appear as early divergent outgroups (Figure 1A).

In addition to the lengthy interdomain sequences, MAST kinases are also distinct within the AGC family due to their characteristically large activation segments (T-loops, 37 amino acids long in MAST1-4 and 460 amino acids long in MASTL) [37,54]. T-loops are crucial components within the kinase catalytic core that determine the activation state, commonly referred to as DFG^in^ (active)/DFG^out^ (inactive) states [55], and confer substrate specificity [56]. Investigating the development of the T-loop could provide mechanistic insights into evolutionary parallel pathways that regulate the specificity and activation of the MAST and MASTL kinase lineages relevant to therapeutic development.

Focusing on T-loop sequences indicates that the longer and more disordered activation segments from early eukaryotes (DDB G0272282, B3RKI6, IREH1, pkgA) are intermediate in length between mammalian MAST and MASTL paralogs (Figure 1A). This is consistent with the observation that protists, plants, and some animals (kin-4 in worms) do not have separate MAST and MASTL lineage kinases. We concluded that the orthologous kinases in earlier eukaryotes, like protists and plants, represent MAST proteoforms before diverging into separate MAST and MASTL paralogs. Thus, the primordial MAST kinase originally had a T-loop of intermediate length that later diverged into two lineages around the time of plant and early animal divergence: one that preferentially lengthened the T-loop even further (MASTL, Figure 1C) and the other that shortened and stabilized it (MAST, Figure 1C).

**Figure 2:**
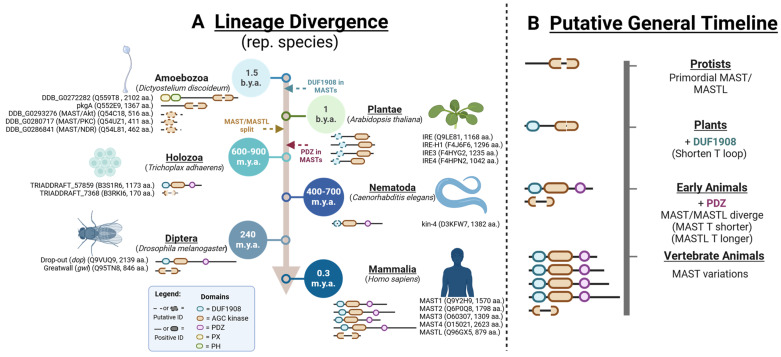
MAST kinase evolution is marked by domain expansion and diversification. (**A**) Detailed lineage divergence with representative species of MAST/MASTL kinases in evolutionary history. Identified species with positive (solid) or putative (dashed) identified sequences or domains indicated with accession numbers. (**B**) General Summary of the MAST kinase evolution timeline. Protein cartoons are representative, with corresponding significant events accordingly outlined. The graphics were created in BioRender. Lemke, M. (2025) https://BioRender.com/x88u478, accessed on 29 March 2025.

The progression of MAST domain conservation and T-loop divergence outlines four significant events in eukaryotic evolutionary history (Figure 2). The presence of a putative DUF domain in plants suggests that the emergence of this domain occurred in an ancestor shared with animals, along with the development of shorter T-loops. The MAST/MASTL divide likely happened around the time of early divergent animals due to the absence of defined MAST and MASTL niches in eukaryotes before *Trichoplax* (Figure 2). At this juncture, we also observe the emergence of the PDZ domain in the MAST sequence, as is evident from the *Trichoplax* MAST ortholog. The shorter T-loop MAST lineage then presumably underwent several gene duplication events in the development of vertebrate animals, leading to multiple isoforms of MASTs. This might suggest a selective advantage of multiple MASTs and further functional diversification, possibly due to tissue specificity in more complex organ systems.

## 4. MAST Kinase Expression and Interactomes

MASTs are differentially expressed across a broad range of human tissues [28,57]. The direct function and substrates of MAST1-4 are all currently unknown, but the tissue specificity may represent some degree of functional exclusivity within the MAST subfamily. We then sought to bioinformatically summarize both the expression patterns and known protein interactors of MAST1-4 using publicly available databases to differentiate their functionality in vertebrate animals.

**Figure 3:**
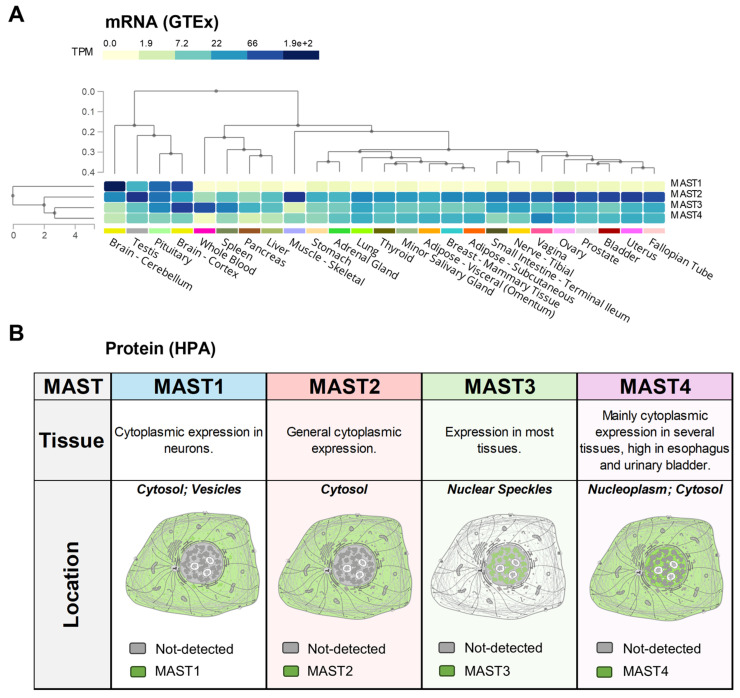
Human MAST kinase expression and localization are heterogeneous. (**A**) Multi-MAST1-4 gene query in the GTEx portal (BROAD Institute, supported by the Common Fund of the Office of the Director of the National Institutes of Health and by NCI, NHGRI, NHLBI, NIDA, NIMH, and NINDS) obtained in December 2024. (**B**) Summary of the curated tissue specificity and subcellular location data from the Human Protein Atlas (HPA, https://www.proteinatlas.org/) for MAST1-4.

**Figure 4:**
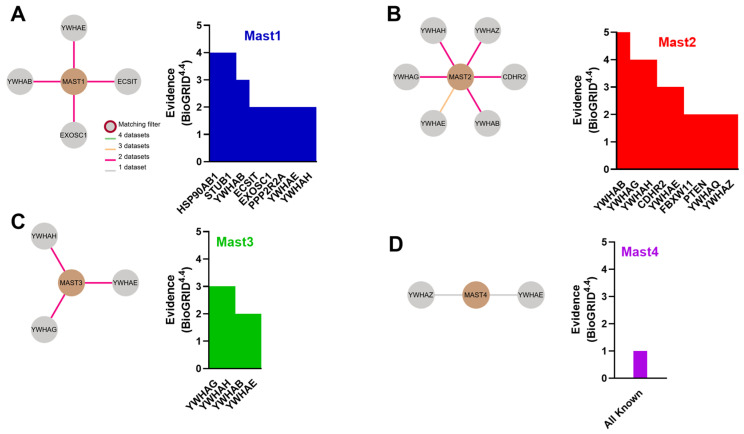
MAST1-4 all likely interact with 14-3-3 proteins. Consensus interaction graphic from HPA summarizing interacting proteins from four interactome databases (IntAct, BioGrid, OpenCell, BioPlex) and manually identified interactions found in BioGrid v4.4. Evidence = number of identifications seen more than once for (**A**) MAST1, (**B**) MAST2, (**C**) MAST3, and (**D**) MAST4. All interactions with MAST4 we identified have only been seen once.

MAST1 is primarily associated with the brain, as transcripts are almost entirely brain-specific (Figure 3A). Localization studies reported in the Human Protein Atlas database suggest that the protein is mainly expressed in the cytoplasm of neurons (Figure 3B). Mutations in MAST1 cause mega-corpus-callosum syndrome with cerebellar hypoplasia and cortical malformations without megalencephaly (MCCCHCM) [58]. MAST1 has also been implicated as a target for cancer therapy since its upregulation can often lead to cancer [28]. Direct inhibition, or destabilization of MAST1 by inhibiting heat shock protein (HSP) and ubiquitin-specific processing (USP) proteins, has been shown to rescue chemotherapeutic sensitivity in vivo [59,60,61,62]. Other interactions have been observed between MAST1 and 14-3-3 proteins (Figure 4A). MAST to 14-3-3 protein interactions have been noted with MAST2-4 as well, with slight variations in the 14-3-3 isoform (Figure 4A–D).

By comparison, the other MAST kinases are much more broadly expressed. MAST2 (originally MAST205 [63]) was the first MAST discovered and identified from the spermatid manchette’s microtubule fraction. While MAST2 transcripts are highest in gonadal tissues and skeletal muscle (Figure 3A), the protein is broadly expressed in the cytoplasm of most human cells (Figure 3B). MAST2 upregulation is permissive to cancer progression, whereas point mutations have been linked to cardiovascular disease and type 2 diabetes mellitus [28]. MAST2 gene duplications have been associated with non-obstructive azoospermia [64]. MAST1-3 have all been shown to phosphorylate and stabilize the phosphatase and tensin homolog (PTEN) tumor suppressor [34,44,45,65] (Figure 4C), which has also potentially interacted with MAST4 [66].

MAST3 transcripts are the highest in the brain, whole blood, pituitary, and spleen (Figure 3A). MAST3 mRNA is heterogeneously expressed in the brain and predominates in the cortex [57]. Interestingly, MAST3 is reported to be largely excluded from the cytoplasm and localizes to nuclear speckles (Figure 3B). Like MAST1, point mutations in MAST3 are often associated with neuronal diseases—specifically, developmental and epileptic encephalopathy [67,68]. Other than 14-3-3 isoforms (Figure 4B), MAST3 regulates PP2A in neuronal cells through the only known MAST3 substrate, cAMP-regulated phosphoprotein of molecular weight 16 kDa (ARPP-16) [69,70]. ARPP-16 is a splice variant of ARPP-19, which are both paralogs of the MASTL substrate ENSA, and when phosphorylated (S67 in ENSA, S62 in ARPP-19), they potently inhibit PP2A-B55 to counter CDK1-directed mitotic entry [41,42,69,70,71,72].

Comparatively, MAST4 is the lowest expressed MAST kinase across human tissues (Figure 3A). MAST4 protein is detectable in the cytoplasm of several soft tissues, including the brain, esophagus, and bladder (Figure 3B). MAST4 upregulation and point mutations have also been linked to cancer and childhood epilepsy [28]. However, MAST4 has notable associations with bone differentiation, osteolytic lesions, and multiple myeloma [66,73,74]. Not only does estrogen signaling up-regulate MAST4 expression in bone, but increased MAST4 activity is protective against multiple myeloma, as it drives mesenchymal stromal cell differentiation into bone and cartilage through phosphorylation and subsequent degradation of Sox9 (S494). MAST4 was also recently shown to regulate Tctex-1, or dynein light-chain Tctex-type 1 (DYNLT1), at the primary cilium of mammalian cells, where it accelerated ciliary resorption and is a potential therapeutic target for ciliopathies [75]. However, all other interactions with MAST4 have only been reported once (Figure 4D).

**Figure 5:**
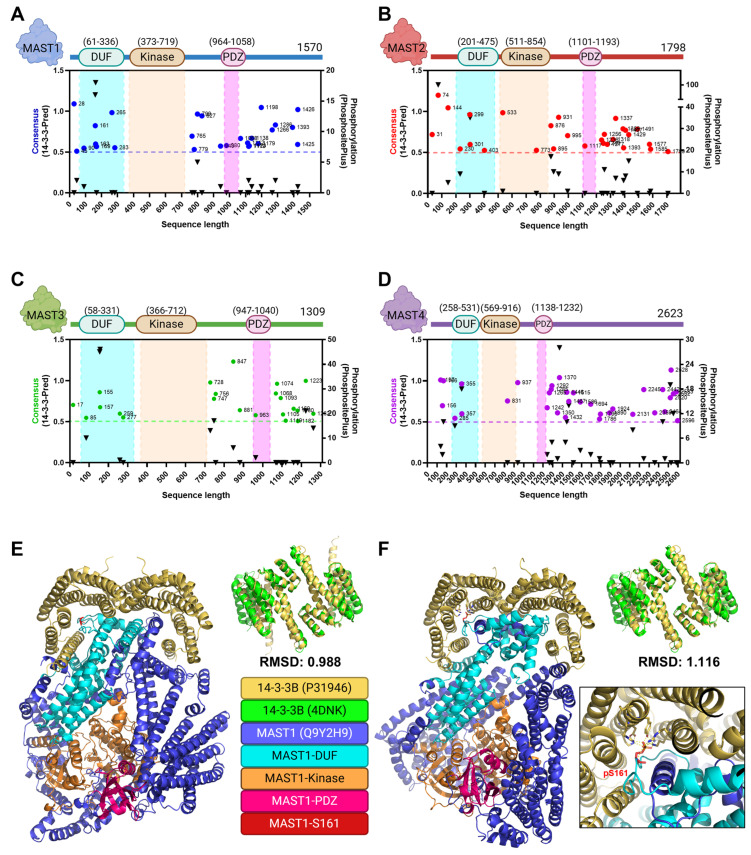
The DUF domain could interact with 14-3-3 proteins. The 14-3-3-Pred consensus scores greater than or equal to 0.5 (dashed line [78]) of residues and a corresponding number of entries in PhosphoSitePlus (v6.7.5) in full-length sequences of (**A**) MAST1, (**B**) MAST2, (**C**) MAST3, and (**D**) MAST4. DUF (cyan) kinase (orange) and PDZ (pink) domain regions are highlighted in the sequence graphs. (**E**) The unmodified sequence of MAST1 (Q9Y2H9) and two copies of 14-3-3β (P31946) (RMSD to 4DNK shown) were loaded into the AlphaFold3 server (https://alphafoldserver.com/). MAST1 interspersed regions (blue), as well as DUF1908 (cyan), AGC kinase (orange), and PDZ (pink) domain architecture, are highlighted. MAST1-S161 is indicated (red). (**F**) As per (**E**), but with S161 modified to phosphoSer-161 (red). The graphics were created in BioRender. Lemke, M. (2025) https://BioRender.com/q81h296, accessed on 29 March 2025.

Due to the strength of the 14-3-3 interaction signal from the interactome databases, we sought to identify the regions in MAST kinases that could interact with 14-3-3 proteins. 14-3-3 proteins typically bind to phosphorylated proteins to regulate protein stability, inter-protein interactions, or localization [76]. Previous analysis of the phosphoproteome of 14-3-3 interactions revealed that MAST2 was a robust phospho-dependent partner of 14-3-3 proteins, but the phosphorylated residues were not identified [77]. We sought to scan MAST kinases for the highest likelihood 14-3-3 target sites, using the 14-3-3-Pred webserver [78], correlated with known phosphorylation events reported in PhosphoSitesPlus [79]. We noted that the frequency of potential binding sites is strikingly high in MAST kinases, as most 14-3-3 interactions described in the literature only operate via a couple of phosphorylated residues [80]. MAST kinases contain numerous Ser/Thr residues reported as phosphorylated [28]. Although many high-scoring 14-3-3 interaction sites were predicted in the C-terminal sequences of MAST1-4, we observed that the N-terminal DUF domain from all MAST kinases also contained several 14-3-3 binding motifs that overlapped with repeatedly seen phosphorylation events (Figure 5A–D).

The sequences containing the conserved domains of MAST1-4 are only about half of the total protein sequence. The rest of the protein sequence, as well as the N-terminal portion of the DUF domain [28], is currently considered an unstructured or intrinsically disordered region (IDR, InterPro, MobiDB [81]). Not only does this make it challenging to obtain a structure of the entire protein, but it could limit future attempts to solve the structure of protein regions that span more than one domain. Fortunately, developing trained modeling software like AlphaFold facilitates predicting how these molecules might fold in nature [23]. AlphaFold models are trained based on a deep machine-learning methodology derived from primary multiple sequence alignment (MSA) and curated structures from the protein data bank (PDB). This provides a powerful tool for discerning protein functionality through structural dissection that, as described above, was impossible with existing methods [82,83]. Therefore, these models can be used to discern MAST kinase molecular mechanics, model drug interactions, and explain why specific point mutations are causal in human disease.

Using the open-access AlphaFold web server, we sought to predict the interaction with 14-3-3β and MAST1 as proof of concept. 14-3-3β was chosen due to the overlapping and strong frequency signal in multiple interactome databases with MAST1-3. MAST1 was chosen as representative of the MASTs due to the median sequence length of intrinsically disordered portions in the range of MAST kinase sizes (879-2623 aa.). AlphaFold3 was used to generate a model of the 14-3-3β dimer similar in structure to a solved 14-3-3β dimer (4DNK) (Figure 5E,F) [24]. Interestingly, the 14-3-3β binding pockets were oriented toward Ser residues around the DUF domain (Figure 5E), specifically around the serine residue at position 161 in the DUF domain (S161). Since 14-3-3 interactions are largely phosphorylation-directed, we then used the PTM capabilities offered by the server to generate the same model with phospho-S161, one of the highest-scoring and highest phosphorylation frequency residues in the MAST1 sequence (Figure 5A). As expected, phosphorylation of Ser161 improved the interaction between the 14-3-3β binding pocket and the DUF domain via localization of polar interactions with the phosphate group (Figure 5F). This may imply that the DUF domain could partially serve as a 14-3-3 binding site.

## 5. Point Mutation In Silico Analysis

In silico predictions of the stability consequences of protein point mutagenesis have been previously described [25,26,27,84]. Protein folding is driven by energetic deposition in the topology of the tertiary structure and is influenced by molecular interactions and modifications between residues. The effects of mutations relative to the wild type within that landscape are therefore calculable through Gibbs free energy (ΔΔG) estimations. While not definitive, these predictive calculations offer potential explanations for the in vivo consequences of mutations. Using these estimations, we sought to dissect the existing AlphaFold-generated MAST models for conserved and novel regulatory mechanisms within MASTs relative to AGC kinases. We performed structure-based in silico point mutagenesis of identified disease-causal protein variants of MAST kinases to gather functional insights into disease progression.

**Figure 6:**
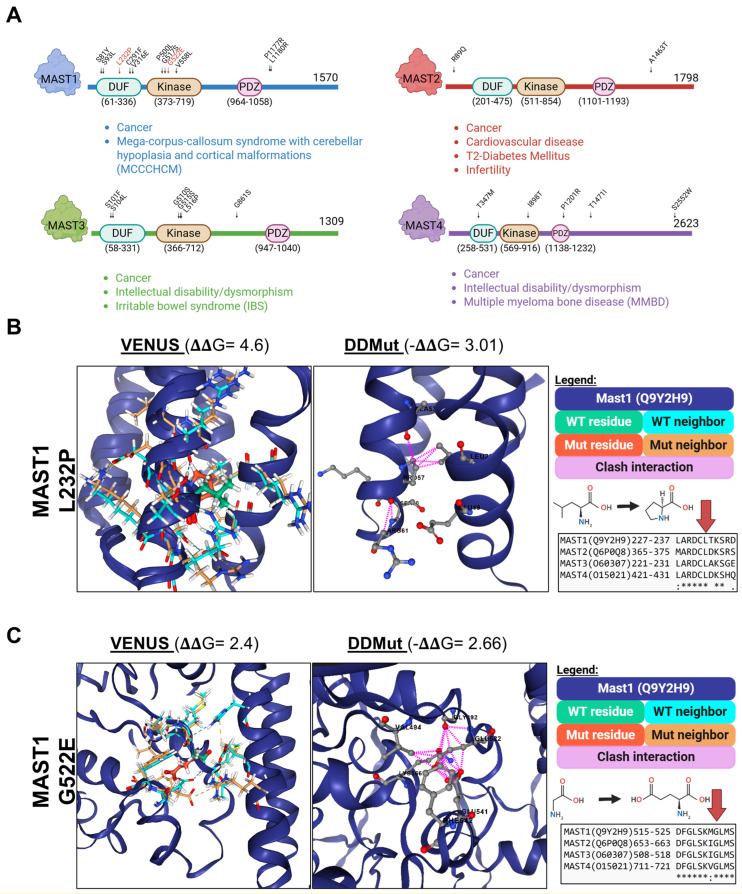
In silico prediction of MAST kinase point mutations. (**A**) Visualization of reported pathogenic MAST mutation’s location in each respective kinase. The overall disease associations for each kinase are listed below each cartoon. (**B**) Demonstration of the consequences of the MAST1 L232P mutation in the DUF domain of MAST1 (2M9X). The prediction and structural representation of the same mutation from both VENUS (left) and DDMut (middle). The MAST1 sequence (dark blue) of the wild-type residue (green) and its neighborhood (light blue), as well as the mutant residue (dark orange) and its neighborhood (orange), are indicated, as well as the predicted residue interaction clashes (DDMut, pink). (Right bottom) Outlined sequences of the DUF domains of MAST1-4 aligned by Clustal Omega (1.2.4) MSA. (**C**) As per (**B**) but with MAST1-G522E. (Right bottom) Outlined sequences of the T-loops of MAST1-4 aligned by Clustal Omega (1.2.4) MSA. Asterisks are explained in Table 1. The graphics were created in BioRender. Lemke, M. (2025) https://BioRender.com/w30g516, accessed on 6 April 2025.

Generally, ΔΔG values of single-point mutations ± 1 kcal/mol are considered significant [85]. Taking a conservative perspective, our threshold for significance was set at ΔΔG ± 2 and was agreed upon by two freely available ΔΔG web calculators, Variant Effect oN Structure (VENUS [25,26]) and DDMut [27], to garner consensus on the effects of each point mutation. Significant ΔΔG from a single site was only considered a possible determination. Taking reported pathogenic mutations in MAST1-4 [28,46,47,48], we outline the location and predicted effects on disease mutants (Table 1). Interestingly, most MAST pathogenic variants were in the DUF and catalytic domains (Figure 6A). MAST2 and 4 mutations were sparse and primarily localized to the predicted IDR. However, MAST1 and MAST3 showed highly similar mutational profiles, especially at the start of the T-loop (DFG) (Table 1).

Most missense mutations were predicted not to affect protein stability (Table 1). We interpreted the results of these mutations as changes in the regional PTM topology contributing to functional dysregulation [11,79,86]. However, two mutations in MAST1—L232P in the structured C-terminal portion of the DUF domain and G522E (DFG^+5^)—had significantly destabilizing consensus scores. Both residues are conserved in all four MASTs (Figure 6B,C). The L232P destabilizing mutation makes biomechanical sense because the unique cyclical side chain of proline often ends alpha-helical structures, as opposed to leucine, one of the best alpha-helix stabilizers [87]. In the case of G522E, glycine is the most compact side chain, and substitution with the polar and negatively charged glutamate could hinder the stabilization of the DFG^in^ conformation and likely result in activity downregulation.

When viewed in the structure for the DUF domain (Figure 6B) and the model of the kinase domain (Figure 6C), it is evident that interactions in the wild-type neighborhood are perturbed in the mutated state (clash interactions). L232P destabilizes the side chain interactions with nearby Ala, Leu, and Arg residues, likely resulting in DUF domain misfolding (Figure 6B). G522E is in an interesting position at the apical start of the T-loop, near what is known as the N-terminal anchor—a stabilized motif characteristic of the active conformation [88]. The glutamate substitution dramatically alters the ability of the N-terminal anchor to pack correctly next to adjacent valine, phenylalanine, and nearby secondary glutamate due to steric and electrostatic clashes (Figure 6C). Based on this model, this substitution likely limits the ability of the kinase to enter the DFG^in^ conformation [54,89] and probably dysregulates MAST1 kinase activity in vivo.

## 6. Conclusions

Here, we present an analysis of the knowledge base of MAST kinases’ development, diversification, and functionality integrated with insights from predictive deep-learning software and available literature on pathogenic mutations. Based on primary sequence and structural homology, we conclude that protist and plant MAST paralogs demonstrate a more primitive morphology with elements of the separate MAST and MASTL kinases found in more recently divergent species. This is seen in the intermediate length of the T-loop insertion and the absence of the DUF and PDZ domains found in animal MAST lineage kinases. Plants were likely among the first eukaryotes to develop the DUF domain, albeit highly divergent from that in animals.

The themes of domain expansion and diversification that we see in the MAST lineage are standard in eukaryotic protein evolutionary history [90,91]. Therefore, organisms with complex tissue and organ systems may differentially express the MAST kinases to diversify MAST kinase functionality, which is evident in human expression profiles. However, the predominant interactome signal that unifies the MASTs is phosphorylation-dependent regulation by 14-3-3 proteins, possibly around residues in the DUF domain. It is unclear exactly how 14-3-3 proteins impact MAST activity, but research shows that 14-3-3 proteins inhibit and sequester RAF kinases [92,93], as well as maintain the activation of CaMKK2 [94]. Further direct experimentation, as has been carried out with MAST2 [77], is needed for definitive conclusions.

Structural predictions offer a starting point for future hypotheses and independent experimental validation. The predictive models summarized here provide a tentative basis for investigating MAST kinase regulation and drug discovery. We estimated that only a few pathogenic mutations will likely impact stability (Table 1, Figure 6). The others, often at common sites of PTM, could represent alterations to regulatory inputs into MAST kinases, leading to activity dysregulation [95]. The mutations predicted to be destabilizing will need to be verified more accurately through experimental methodologies, such as thermal shift assays or mass spectrometry-based experimental approaches [96,97].

Altogether, our work serves as a call to action for further investigation into MAST kinases. With multiple disease associations and many yet unanswered basic questions outlined above open for investigation, the MAST kinases represent a treasure trove of novel therapeutic opportunities. This outline will hopefully better orient researchers and clinicians in their efforts to understand the structural biology and the basis for diseases that currently burden many patients [28].

## Figures and Tables

**Table 1:** MAST kinase point mutation ΔΔG estimation and effect interpretation.

Isoform	Mutation, Disease [28,46,47,48]	Location	ΔΔG * (kcal/mol) (VENUS)	-(ΔΔG) ** (kcal/mol) (DDMut)	Interpretation
MAST1 (Q9Y2H9)	S81Y, cancer S93L, neuronal disability	DUF DUF	−0.7 −1.8	−0.05 −0.06	Neutral, altered phosphosite Neutral, loss of phosphosite
	L232P, MCCCHCM C291F, cancer	DUF DUF	4.6 0.1	3.01 0.48	Destabilizing Neutral, loss of di-sulfide bond
	V316E, cancer P500L, neuronal disability G517S, MCCCHCM G522E, MCCCHCM V558L, MCCCHCM P1177R, neuronal disability L1180R, neuronal disability	DUF Catalytic-HRD^+3^ Catalytic-G of DFG Catalytic-DFG^+5^ Catalytic-APE^+1^ IDR IDR	−0.1 0.6 −0.9 2.4 0.7 0.3 −0.2	0.47 0.93 0.06 2.66 0.55 −0.07 −0.06	Neutral, altered hydrophobicity/Ub site Neutral, altered RD pocket flexibility Neutral, may stabilize DFG^in^ Destabilizing Neutral, may alter DFG^in^/DFG^out^ shift Neutral, altered electrostatics Neutral, altered electrostatics
MAST2 (Q6P0Q8)	R89Q, vascular disease A1463T, TII-diabetes	IDR IDR	−2.3 0.3	0.21 0	Possibly stabilizing Neutral, gain of phosphosite
MAST3 (O60307)	S101F, neuronal disability	DUF	−9.8	−0.05	Possibly stabilizing
	S104L, neuronal disability G510S, neuronal disability	DUF Catalytic-G of DFG	0.7 −1.0	−0.06 0.16	Neutral, loss of phosphosite Neutral, may stabilize DFG^in^
	G515S, neuronal disability	Catalytic-DFG^+5^	−0.3	0.66	Neutral, may alter DFG^in^/DFG^out^ shift
	L516P, neuronal disability G861S, IBS	Catalytic-DFG^+6^ IDR	−0.3 −4.4	0.59 −0.04	Neutral, may alter DFG^in^/DFG^out^ shift Possibly stabilizing
MAST4 (O15021)	T347M, neuronal disability I898T, neuronal disability	DUF Catalytic-HM motif	−1.6 0.3	−0.01 1.37	Neutral, loss of phosphosite Neutral, reduced activity
	P1201R, neuronal disability T1471I, neuronal disability S2552W, neuronal disability	PDZ IDR IDR	1.2 −2.7 0.7	−0.15 −0.07 −0.05	Neutral, altered PDZ flexibility Possibly stabilizing Neutral, loss of phosphosite

* = Positive ΔΔG > 2 kcal/mol: Indicates a destabilizing mutation; Negative ΔΔG < −2 kcal/mol: Indicates a stabilizing mutation. ΔΔG estimations shown with backbone movement allowed. ** = VENUS reports positive ΔΔG as destabilizing (Mut ΔG–WT ΔG); DDMut reports negative ΔΔG as destabilizing (WT ΔG–Mut ΔG). For comparison, DDMut values will be reported as -(ΔΔG).

## Data Availability

These data were derived from the following resources available in the public domain: UniProt (https://www.uniprot.org/); InterPro (https://www.ebi.ac.uk/interpro/, accessed on 31 March2025); Genotype-Tissue Expression project (GTEx, https://www.gtexportal.org/home/); Human Protein Atlas (HPA, https://www.proteinatlas.org/); Biological General Repository for Interaction Datasets (BioGRID, https://thebiogrid.org); PhosphoSitePlus (https://www.phosphosite.org/homeAction); 14-3-3-Pred (https://www.compbio.dundee.ac.uk/1433pred/, accessed on 3 December 2024); AlphaFold3 (https://alphafoldserver.com/welcome); MichelaNGLo-VENUS (https://venus.sgc.ox.ac.uk/venus); and DDMut (https://biosig.lab.uq.edu.au/ddmut/, accessed on 29 November 2024). The original contributions presented in this study are included in this article. Further inquiries can be directed to the corresponding author.
